# The black hole of the transition process: dropout of care before transition age in adolescents

**DOI:** 10.1007/s00787-021-01939-8

**Published:** 2022-01-20

**Authors:** Blanca Reneses, Almudena Escudero, Nuria Tur, Luis Agüera-Ortiz, Dolores María Moreno, Jerónimo Saiz-Ruiz, Mayelin Rey-Bruguera, Maria-Fuencisla Pando, Maria-Fe Bravo-Ortiz, Ana Moreno, Ángel Rey-Mejías, Swaran P. Singh

**Affiliations:** 1 Institute of Psychiatry and Mental Health, Instituto de Investigación Sanitaria (IdISSC), San Carlos University Hospital, Av. Profesor Martín Lago s/n, 28040 Madrid, Spain; 2 Centro de Investigación Biomédica en Red de Salud Mental (CIBERSAM), Complutense University, Madrid, Spain; 3 Department of Psychiatry, Instituto de Investigación (I+12), Hospital Universitario 12 de Octubre, Madrid, Spain; 4 Institute of Psychiatry and Mental Health, Gregorio Marañón University Hospital, Madrid, Spain; 5 Alcalá de Henares University, Alcalá de Henares, Spain; 6 Centro de Investigación Biomédica en Red de Salud Mental (CIBERSAM), Madrid, Spain; 7 Hospital Universitario Ramón Y Cajal. Servicio de Psiquiatría, Madrid, Spain; 8 Department of Psychiatry, La Paz University Hospital, Instituto de Investigación IdiPaz, Madrid, Spain; 9 Centro de Investigación Biomédica en Red CIBERSAM, Autónoma University, Madrid, Spain; 10 Department of Psychiatry, Príncipe de Asturias University Hospital, Alcalá de Henares, Madrid Spain; 11 Complutense University, Departamento de Psicobiologia Y Metodología en Ciencias del Comportamiento, Madrid, Spain; 12 Health Sciences Warwick Medical School, University of Warwick, Coventry, UK

**Keywords:** Adolescent psychiatry, Patient dropout, Transitional care, Continuity of care, Care pathways

## Abstract

Recent evidence confirms the risks of discontinuity of care when young people make a transition from child and adolescent mental health services (CAMHS) to adult mental health services (AMHS), although robust data are still sparse. We aimed to identify when and how patients get lost to care during transition by tracking care pathways and identifying factors which influence dropping out of care during transition. This is a retrospective observational study of 760 patients who reached the transition age boundary within 12 months before transition time and being treated at CAMHS for at least during preceding 18 months. Data were collected at two time points: last visit to CAHMS and first visit to AHMS. Socio-demographic, clinical and service utilization variables on CAMHS treatment were collected. In the 12 months leading up to the transition boundary, 46.8% of subjects (*n* = 356) withdrew from CAHMS without further contact with AHMS, 9.3% withdrew from CAHMS but were referred to AHMS by other services, 29% were transferred from CAHMS to AHMS, 10% remained at CAHMS and 5% patients were transferred to alternative services. Fifty-six percent of subjects experience cessation of care before the transition age. The risk of dropout increases with shorter contact time in CAMHS, is greater in subjects without pharmacological treatment, and decreases in subjects with psychosis, bipolar disorder, eating disorders, mental retardation, and neurodevelopmental disorders. This study confirms that a large number of people drop out of care as they approach the CAMHS transition and experience discontinuity of care during this critical period.

**Supplementary Information:**

The online version contains supplementary material available at 10.1007/s00787-021-01939-8.

## Introduction

The importance of prioritizing adolescent mental health care is supported by the fact that most mental disorders appear before the age of 25 [1] and their contribution to the years lived with disabilities is 25% between 0 and 24 years of age [2]. However, services often do not meet the mental health needs of adolescents [3], with transition between child and adolescent mental health services (CAMHS) and adult mental health services (AMHS) being an important contributor to this unmet need when they reach the age of transition between both [4, 5].

The phenomenon of the transition between CAMHS and AMHS has been an increasing reason for study in recent years. The seminal TRACK study in the UK [6] commenced such research in the EU, highlighting the existence of a discontinuity of care during the transition in 50% of cases. Studies in other countries with advanced health systems have also shown similar risk of patient loss and system fragility during this period [1, 5, 7], suggesting a significant public health problem [8].

The available data suggest that patients at higher risk of being lost during the transition process are those with apparently less serious disorders, while subjects with psychotic, bipolar, eating, or neurodevelopmental disorders are more likely to make an effective transition [6] and to have a more adequate care pathway in terms of access to mental health services [9].

A comprehensive study (MILESTONE study) on the phenomenon of transition was recently conducted in eight EU countries, which includes objectives related to the knowledge of organizational policies and structures, the development of tools to support the transition, and ethical recommendations [8, 10]. Its results must be contextualized within the organization of service provision in each territory to accurately analyze care in the transition stage and seek effective solutions in terms of health policies [11, 12]. Furthermore, to date no specific data on adolescent care pathways in the pre- and post-transition stage are available in sufficiently large samples of patients within a given territory.

This study (CRECER Project) has been developed to answer these questions and has the following objectives:

1. To identify care pathways during the transition process and gaps through which patients may be lost to care.
2. To identify the components of the care pathway of patients that reach AMHS along the first 6-month period of treatment there.
3. To determine factors predicting being lost to care during the transition process.
4. To evaluate the quality of the transition process within a significant region of Spain (Madrid).

The methods and results of objectives 1 and 3 are presented in this article.

## Methods

### Design

This is an observational retrospective study of a representative sample of patients in a well-defined geographical area.

### Location of the study and health-care organization framework

The study was conducted at seven highly specialized public general hospitals and their catchment care areas in Madrid (Spain) from January 2017 to March 2018 including 12 CAHMS and 18 AHMS. They cover a population of 2.4 million people who live in urban, metropolitan, and rural geographic sectors from diverse socioeconomic backgrounds. These services can be considered representative of the public care system in other Spanish regions, as all of them share the basic principles of health-care organization.

Spain has a public health-care system with universal coverage. Care is sectorized into catchment areas with two levels: “primary care” and “specialized and hospital care”. CAMHS and AMHS are at the “specialized and hospital-care level”. Both services usually belong to the same department of psychiatry and mental health, so that patients seen at CAHMS can be referred to AHMS directly, without the need for a previous step through primary care.

Every hospital has one or more outpatient CAMHS. The age of transition for mental health care is 18 years. Transition between CAMHS and AMHS is not protocolized at a regional level, so every CAMHS refers to its specific AMHS in different ways. Usually, patients have to request a new appointment in the corresponding AMHS through a referral sheet. The primary care step is not necessary in this process because it is a referral between specialized care services.

### Sample

We identified a cohort of service users approaching the CAMHS/AMHS boundary at the aforementioned hospitals. These subjects would have reached the age of 18 years within a 12-month period between 2015/07/01 and 2016/07/31 and had been treated at CAMHS during the preceding 18 months (between 2015/01/01 and 2016/07/31).

The inclusion criteria were: being born between 1997/07/01 and 1998/07/31, having attended CAMHS at least once between 2015/01/01 and 2016/07/31 (namely, before the age of transition), and either not having been discharged at the CAMHS (that is, continuing care at CAMHS) or having been referred directly from CAMHS to an AMHS. The exclusion criteria were: having been discharged at CAMHS before the transition age, having been referred to another hospital not included in the study, or having moved out of the geographical areas included in the study.

**Fig. 1:**
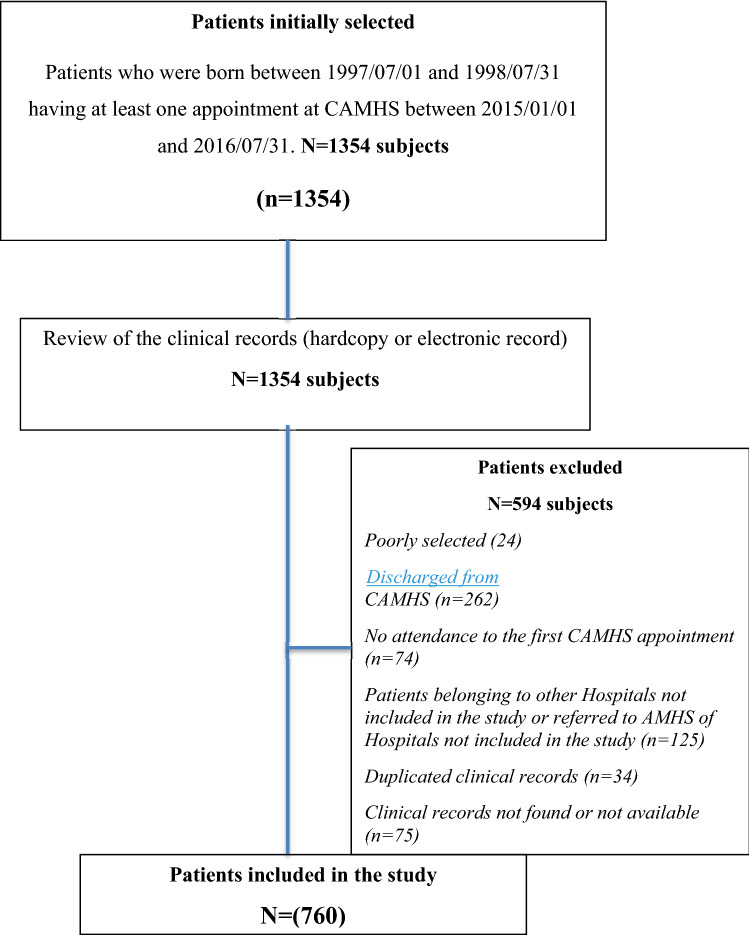
Case ascertainment to sample selection

The case ascertainment flowchart is explained in Fig. 1.

### Procedure for extracting data from the clinical records

Two researchers (clinical psychologists) were trained in clinical data extraction in a pilot study of 90 cases. Clinical records (electronic or hardcopy) were manually reviewed and data extraction was anonymized at the origin.

To avoid possible duplication, subjects’ records were identified at the end of the data collection phase. Possible duplicate records were identified and included as one case.

### Data collection time points and variables

Study data were collected and managed using REDCap electronic data capture tools [13]. Retrospective data collection was conducted at four time points alongside every clinical record: T0: last visit at CAMHS; T1: first visit at AMHS; T2: 3 months after T1; T3: 6 months after T1.

Socio-demographic variables were collected in T0.

Clinical and psychosocial variables included principal and secondary diagnosis at the last visit to CAMHS (T0), duration of care at CAHMS, substance abuse, prescribed pharmacological and psychosocial treatment at all time points, and all variables related with the use of mental health-care services before T0 and in the period between T0 and T3.

Variables related to the quality of the transition process included transfer of information, a period of joint care between CAMHS and AMHS, specified transition plan, long-term continuity of care plans (continuity of care 3 or 6 months after transition or without continuity) and waiting time between T0 and T1.

Variables related to pathways during transition were emergency room visits between T0 and T1, lost appointments between T0 and T1, discharges during periods T1–T3, and follow-up care during the 3- and 6-month periods (T1–T2 and T2–T3) (number of appointments, day hospital admissions, hospital admissions to psychiatric units, and emergency room visits).

Data were obtained from two sources: patient’s clinical records in electronic or hard copy formats for clinical data, and hospital information systems for data related to CAMHS and AMHS appointments, withdrawal appointments, origin of appointments, emergency room visits, day hospital admissions, and psychiatric hospitalization admissions.

### Ascertaining principal and secondary diagnoses

Diagnoses were extracted from clinical records. All diagnoses were transformed to ICD-10 according to the official conversion tables. Diagnoses were collected just at the time of referral to AMHS or during the last visit to CAHMS.

Given the large number of diagnoses and to facilitate their comparability, these were grouped into eight categories according to the grouping criteria used in the TRACK study [6] with a modification in the “neurodevelopmental disorders” diagnostic group, which was divided into “ADHD” and “severe neurodevelopmental disorders”.

Grouped diagnostic categories:

- Psychosis, bipolar disorders and depression with psychotic symptoms (ICD 10 codes: F20–29; F30–F31; F34.0; F32.3; F33.3; F12.5; F19.5).
- Mood, anxiety, stress-related disorders, somatoform disorders, and obsessive–compulsive disorder (ICD 10 codes: F32; F33; F34.1; F38; F40- F45; F48; F93; F94; F98; F99).
- Eating disorders (ICD-10 codes: F50).
- Mental retardation and neurodevelopmental disorders excluding attention deficit and hyperactivity disorder (ADHD) (ICD codes: F70–79; F80; F83; F84; F88).
- ADHD (ICD code F90).
- Substance misuse disorders (ICD-10 codes F10-19, excluding psychotic disorders due to use of toxic substances).
- Behavior disorders (F91; F92).
- Emerging personality disorders (F60; F61; F63; F64; F66).

A detailed description of ascertaining and grouping diagnosis is explained in Annex I and II (additional materials).

### Care pathway definitions

The care pathways followed by the subjects after their last visit to CAMHS were defined using the following variables: referral from CAMHS to AHMS, existence of an appointment at AMHS 6 months after the last appointment at CAMHS, attendance or not at the first appointment at AHMS, referral from other services to AHMS (this information is specified in the medical records), absence from appointments at CAMHS after the last attendance recorded in the medical record, and continuation of care at CAMHS after the transition age.

### Dropout definition

For the purposes of this study, a patient is considered to have dropped out when he/she was not discharged from CAHMS and missed his/her appointments at CAMHS after the last registered attendance. It also included patients not discharged but who stopped attending the CAMHS after the last registered attendance, but had not a formal appointment (in some cases requesting for a formal appointment depends on the patient or family after the therapist’s recommendation to do so).

After identifying the care pathways, the subjects were grouped according to whether or not they had dropped out of the CAMHS. Included in the dropout group were those subjects who had initially withdrawn from the service, but had been seen at AMHS in the following 6 months for being referred by other services (primary care or emergency).

### Statistical analysis

Firstly, qualitative and quantitative variables were summarized.

Secondly, we performed a preliminary analysis (Table 1) to compare patients who dropped out from the care system before transition with those who did not, regarding demographic and clinical variables. In this analysis, a contrast of independence of Chi^2^ was used for qualitative variables. For quantitative variables we performed a Mann–Whitney *U* test for non-parametric analyses reporting in results the more standardized *U* *Z*-scored, because none of our variables satisfied the assumptions of the general lineal model (GLM).

**Table 1:** Comparative analysis between groups who dropped out and did not drop out of care according to their socio-demographic characteristics and service utilization variables before transition

	Total sample (*n* = 760)	Patients who withdrew from CAMHS (*n* = 426)	Patients who did not withdraw (*n* = 334)	Comparison statistic	Cohen’s *d*
Female, *n* (%)	402	239 (59.5)	163 (40.5)	$\chi_{1}^{2}$ = 4.005 (*p* = **0.045**)^a^	0.146
Place of birth: Foreign national, *n* (%)	135	87(64.4)	48 (35.6)	$\chi_{1}^{2}$ = 5.258 (*p* = **0.022**)^a^	0.167
Family history of mental health disorders, *n* (%)	257	139 (54.1)	118 (45.9)	$\chi_{1}^{2}$ = 0.283 (*p* = 0.595)	0.039
Protective care, *n* (%)	27	15 (55.6)	12 (44.4)	$\chi_{1}^{2}$ = 0.003 (p = 0.958)	0.004
Police records, *n* (%)	36	20 (55.6)	16 (44.4)	$\chi_{1}^{2}$=0.004 (*p* = 0.951)	0.005
Drug consumption, *n* (%)^b^	94	54 (57.4)	40 (42.6)	$\chi_{1}^{2}$ = 0.085 (*p* = 0.771)	0.021
Previous episodes of hospitalization at CAMHS, *n* (%)	95	32 (33.7)	63 (66.3)	$\chi_{1}^{2}$ = 22.052 (*p* < **0.001**)^a^	0.346
Previous episodes of day hospital treatment at CAMHS, *n* (%)	72	24 (33.3)	48 (66.7)	$\chi_{1}^{2}$=16.666 (*p* < **0.001**)^a^	0.299
One or more psychiatry ER visits 2 years before transition, *n* (%)	140	68 (48.6)	72 (51.4)	$\chi_{1}^{2}$ = 3.899 (*p* = **0.048**)^a^	0.144
Average months of treatment at CAMHS, mean (SD)	29.60 (36.7)	20.70 (29.9)	40.95 (41.2)	*Z*_U_ = 7.629 (*p* < **0.001**)^a^	0.574
Pharmacological treatment, *n* (%)	356	142 (39.9)	214 (60.1)	$\chi_{1}^{2}$ = 71.040 (*p* < **0.001**)^a^	0.642
Social worker intervention (%)	162	95 (58.6)	67 (41.4)	$\chi_{1}^{2}$ = 0.560 (*p* = 0.454)	0.054

Protective care = child taken into protection by the state. Drug consumption = included patients with an established diagnosis and patients with drug consumption notated in their clinical record. *ER* emergency room, *SD* standard deviation. *n* number of patients. *χ*^2^ Chi square. *Z*_*U*_ Mann–Whitney *U* test typified

^a^Statistical significance (95% confidence interval) *p* < 0.05

^b^Drug consumption registered in clinical records or drug abuse or drug dependence as established diagnosis in health records

To attain a predictive model to identify what patient characteristics can be risk factors for withdrawal from care before the transition age, we performed a binary logistic regression (BLR). First, we checked the BLR model assumptions. Second, the candidate predictors used for the BLR model were those that reached statistical significance in the bivariate model (first step). Third, we continued with the dichotomous criterion variable "dropout of care", considered as reference to predict the level of "patients who dropped out before transition". The criterion we used was to extract the variable with the lowest level of significance in adjusting the model, until the best fit was achieved. In each step, we checked the change in coefficients associated with the rest of the variables after exclusion. We would not exclude factors whose coefficient varied more than 20%, because it would have a mediating or confusing role in the model. Finally, a ROC curve was used to analyze the degree of prediction of the model.

All analyses were performed with SPSS Statistics IBM© version 22. We set our confidence interval level as 95%. Effect sizes were reported every time that we found a statistically significant effect by odds ratio or Cohen’s D statistics.

### Ethics approval

The San Carlos University Hospital Research Ethics Committee approved this study. All national and international rules on data protection were followed (Organic Law 3/2018, of December 5, on the Protection of Personal Data and Guarantee of Digital Rights, Regulation (EU) 2016/679 of the European Parliament and of the Council of April 27, 2016 and repealing Directive 95/46/EC (General Data Protection Regulation), Regulation (EU) 2018/1725 of the European Parliament and of the Council of 23 October 2018).

Because this study was anonymized at the origin of data collection, it was declared exempt of informed consent by the research ethics committee.

## Results

Socio-demographic and clinical variables of the whole sample and distribution depending on dropout status are shown in Table 1. Median age was 17.83 years (interquartile range: Pc 75–Pc 50 = 18.2500–17.3333 = 0.9167).

### Patient status after last visit to CAMHS

A total of 222 subjects (29% of the whole sample) were transferred from CAMHS to AMHS, 74 subjects (10%) continued to receive treatment at CAHMS after the age of transition, 300 subjects (39.5%) missed their appointment at CAMHS after the last registered attendance and were not transferred to AMHS, 126 subjects (16.5%) stopped attending the CAMHS after the last registered visit without a new appointment and without a medical discharge and were not transferred to AMHS, and 38 subjects (5%) were transferred to other specialized services outside of the mental health network (i.e., residential facilities for substance abuse disorders).

Subjects who failed their appointments at CAMHS and those who stopped attending CAMHS were unified as a group that dropped out of CAMHS before transition.

### Care pathways

**Fig. 2:**
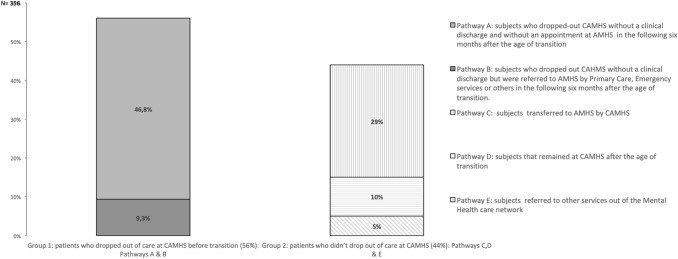
Care pathways after the last visit to CAMHS

Five care pathways were identified from the last visit at the CAMHS. They are described and represented in Fig. 2. These pathways were built taking into account:

- Whether patients were referred to AMHS by a CAHMS professional.
- Whether patients were referred to AMHS by services other than CAMHS.
- Whether there was a last visit at CAMHS registered in the clinical record, but he/she did not have any more visits and had not been discharged.

### Dropout of care at CAMHS before transition

After identifying the five care pathways, the sample was divided into two groups: group 1: patients who dropped out of care at CAMHS before the transition age: pathways A and B; and Group 2: patients who did not drop out of care at CAMHS: pathways C, D and E. These pathways and groups are shown in Fig. 2.

### Differences in socio-demographic and clinical characteristics between groups who dropped out and did not drop out of care before transition

Table 1 shows the comparative analysis between groups who dropped out and did not drop out of care according to their socio-demographic characteristics and service utilization variables before transition.

In the group of patients who dropped out of care, women (*χ*^2^ = 4.1; *p* = 0.04; *D*′ = 0.15) and foreigners (*χ*^2^ = 5.3; *p* = 0.02; *D*′ = 0.17) are more represented. Likewise, less represented are patients without previous episodes of hospitalization (χ^2^ = 22.0; *p* < 0.001); *D*′ = 0.3), without previous episodes of care at day hospital (*χ*^2^ = 16.7; *p* < 0.001; *D*′ = 0.3), without psychiatry ER visits 2 years before transition (*χ*^2^ = 3.9; *p* = 0.04; *D*′ = 0.14), with fewer months of treatment at CAHMS (*Z*_U_ = 7.7; *p* < 0.001; *D*′ = 0.58) and without pharmacological treatment (*χ*^2^ = 71; *p* < 0.001; *D*′ = 0.64).

The differences between groups related to the main psychiatric diagnosis are detailed in Table 2.

**Table 2:** Comparative analysis between groups who dropped out and did not drop out of care according to main diagnosis

	Total sample (*n* = 760)	Patients who withdrew from CAMHS (*n* = 426)	Patients who did not withdraw (*n* = 334)	Comparison statistic	Cohen’s *d*
Psychosis, bipolar disorders and depression with psychotic symptoms (F20–29; F30–F31; F34.0; F32.3; F33.3; F12.5; F19.5)	26	5 (19.2) ^b^	21 (80.8) ^c^	$\chi_{1}^{2}$ = 77.315 (*p* < **0.001**)^a^	0.673
Mood, anxiety, stress-related, and somatoform disorders (F32; F33; F34.1; F38; F40–44; F45; F48; F93; F98; F99)	306	208 (68.0) ^c^	98 (32.0) ^b^		
Eating disorders (F50)	84	38 (45.2)	46 (54.8)		
Mental retardation and neurodevelopmental disorders excluding ADHD (F70–79; F80; F83; F84; F88)	84	25 (29.8) ^b^	59 (70.2) ^c^		
ADHD (F90)	115	58 (50.4)	57 (49.6)		
Substance use disorders (F10–19, excluding psychotic disorders due to toxic substances use)	19	15 (78.9)	4 (21.1)		
Behavior disorders (F91; F92)	16	8 (50.0)	8 (50.0)		
Emerging personality disorders (F60; F61; F63; F64; F66)	53	25 (47.2)	28 (52.8)		
Without a diagnosis (ICD F code) in the clinical record	57	44 (77.2)^c^	13 (22.8)^b^		

*ADHD *attention deficit hyperactivity disorder, *n *number of patients

^a^Statistical significance (95% confidence interval) *p* < 0.05

^b^ 95% Confidence interval standardized residuals (*Z* frequency < 2): cell’s observed frequency is less than the expected frequency

^c^ 95% Confidence interval standardized residuals (*Z* frequency > 2): cell’s observed frequency is greater than the expected frequency

Statistically significant differences of diagnostic categories between groups who dropped out and did not drop out were found (*χ*^2^ = 77.3; *p* < 0.001; *D*′ = 0.67). In the group that dropped out we found an overrepresentation in mood, anxiety, stress-related, and somatoform disorders (68% vs. 32%) and in patients without a diagnosis in the clinical record (77.2% vs. 22.8%). Similarly, we found an underrepresentation in psychosis, bipolar disorders and depression with psychotic symptoms (19.2% vs. 80.8%) and mental retardation and neurodevelopmental disorders excluding ADHD (29.8% vs. 70.2%).

### Prediction of dropout before transition

Results of the logistic regression model are shown in Table 3. The model reached the best fit after six iterations.

**Table 3:** Logistic regression model to identify risk factors to drop out of care before transition

	*B*	Significance	OR (95% CI)
Average months of receiving treatment at CAMHS^a^	−0.011	< **0.001**	0.989 (0.984–0.994)
Pharmacotherapy treatment	−0.951	< **0.001**	0.386 (0.274–0.545)
Diagnostic groups^b^			
Psychosis, bipolar disorders and depression with psychotic symptoms	−1.591	**0.010**	0.204 (0.061–0.683)
Mood, anxiety, stress-related, and somatoform disorders	−0.102	0.770	0.903 (0.455–1.792)
Eating disorders	−0.927	**0.020**	0.396 (0.061–0.683)
Mental retardation and neurodevelopmental disorders excluding ADHD	−1.112	**0.009**	0.329 (0.143–0.756)
ADHD	−0.006	0.989	0.994 (0.447–2.214)
Behavior disorders	−0.895	0.145	0.409 (0.123–1.362)
Substance use disorders	0.300	0.654	1.350 (0.363–5.015)
Emerging personality disorders	−0.831	0.057	0.436 (0.185–1.024)
Without a diagnosis (ICD F code) in the clinical record	Reference	< **0.001**	
Constant	1.433	**0.001**	4.191

*ADHD* attention deficit hyperactivity disorder

Statistical significance (95% confidence interval) *p* < .05 are in bold

^a^The variable has been centralized to the group average: 29.6 months of treatment

^b^Diagnoses have been grouped as described in Table 2

With the final model, four predictors of a higher risk of dropout were identified. These were: shorter duration of treatment at CAMHS [*B* = −0.011; *p* < 0.001; OR = 0.99; 95% OR (0.98–0.99)], not receiving pharmacological treatment [*B* = −0.95; *p* < 0.001; OR = 0.38; 95% OR (0.27–0.54)], not being diagnosed with psychosis, bipolar disorder, depression with psychotic symptoms [*B* = −1.59; *p* < 0.01; OR = 0.20; 95% OR (0.06–0.68)], eating disorder [*B* = −0.93; *p* = 0.02; OR = 0.40; 95% OR (0.06–0.68)], mental retardation or neurodevelopmental disorders (except ADHD) [*B* = −1.12; *p* = 0.01; OR = 0.33; 95% OR (0.14–0.75)].

The final model correctly classified 67.9% of patients with 52.1% sensitivity and 80.3% specificity. To study the predictive value of the regression model for dropout, we performed a ROC curve analysis, which obtained an area under the curve of 0.662 and reduced the mismatch in the null model by 22.2%.

## Discussion

To our knowledge, this is the first study that demonstrates the existence of a serious risk of interruption of care before the transition between a CAMHS and AHMS, thus showing that the problems associated with the transition phenomenon should be considered within a period longer than the one corresponding to the precise moment of the transition between services.

This is a retrospective study of a sample made up of 760 patients treated at CAMHS and potentially transferable to AHMS, and its main objective is to determine when and at what stages patients are lost in the process of transition to AHMS. The most relevant result was that 56% of subjects dropped out from CAMHS immediately before the transition age. Within this group, 9.3% were finally transferred to AMHS by other non-psychiatric services within the 6 months following the transition age. Twenty-nine percent of subjects were transferred to AHMS by CAMHS professionals, 10% continued to receive treatment at CAMHS past the transition age, and 5% were referred to special services outside the psychiatric service network.

Our study partially replicates other previous studies aimed at evaluating the transition process and the associated risk of interruption of care [6, 14]. However, it differs from them in its larger sample size and methodology. In Singh’s study [6], subjects were patients identified by CAMHS professionals as needing transfer to AHMS. Conversely, ours included all potentially transferable subjects, that is, those who, by the age of transition, were being cared for at CAMHS and did not have a medical discharge, similarly to Leavey’s research [14]. Thus, all possible situations were considered before and after referral to AHMS.

The sample composition is similar to that of the previously mentioned studies, both in socio-demographic and clinical characteristics. The high proportion of subjects with a family history of psychiatric disorders (34%) and the low number of subjects with substance use (12.3%) stand out. The fact that the most represented main diagnostic group was that of mood, anxiety, stress-related, and somatoform disorders (40.3%) is consistent with the distribution of morbidity among clinical populations in CAMHS [15] and in the general population [16].

We found that 7.5% of subjects did not have a diagnosis recorded in their clinical records despite being on treatment and not having been discharged. This might be because a small number of professionals could postpone making a formal diagnosis or refuse to use biomedical categories.

### Care pathways

Five care pathways have been identified in the transition process between CAMHS and AHMS. The most frequent (A), which includes 46.8% of the sample, corresponds to subjects who dropped out of services without having been discharged and without an appointment with an AHMS within the 6 months following the transition age. A second pathway (B) includes 9.3% of the sample and corresponds to subjects who also left the service without a medical discharge but who were subsequently referred to AHMS within the 6 months following the transition age. The sum of both groups shows that 56% of young people abandoned follow-up at CAHMS, which is a “black hole” in the care process. This result is relevant because it highlights the risk of losing patients immediately before the potential transition to AHMS.

Different studies show that children and adolescent dropout rates from mental health services at any age range from 28 to 75% depending on the methodology [17]. Different reasons for withdrawal during adolescence have been described, one of which is adolescents’ lack of recognition of their own mental health problems [18] and, hence, their limited search for help. Furthermore, the lack of motivation to seek treatment could be an expression of frustrated expectations of the care system in terms of autonomy, affinity, and competence [19]. On the contrary, the search for help is favored by the clear perception of a benefit from the treatment [20].

Other reasons described for disengagement from services could be the stigma and preference for self-management or the experience of ineffectiveness of the service [21]. The fact that dropout occurs immediately before reaching legal age in our study may suggest that some adolescents initially sought help encouraged by parents [22], but without the personal awareness that it was necessary.

Care pathway C corresponds to subjects who are referred to AMHS from CAMHS and it appears to be a small proportion compared to potentially transferable patients. It is estimated that the proportion of patients in European countries treated at CAMHS who need to be transferred to AMHS ranges from 25 to 49% [23]. In this line, the proportion of our patients transferred is at the lower pole. It is difficult to compare this result with that of other studies since in all of them the samples correspond to subjects previously selected to be transferred by their physicians [6, 24].

There is a group of subjects who remain on CAMHS treatment after the transition age, which accounts for 10% of the sample (care pathway D). This proportion is expected, since it is recommended that the transition be carried out at a flexible time according to the subject’s needs rather than on an administratively set date. It could be interpreted that the transfer was postponed to a time more appropriate for the patient or that there are doubts about the safety of the transition process, or mistrust in the ability of AMHS to adequately attend to the needs.

### Dropout conditioning factors

The group of subjects who left CAMHS before the transition consisted of 426 subjects (56%) compared to a total of 760 in the sample. After studying the possible conditioning factors for dropout, stepwise logistic regression showed that the risk for dropout increases inversely with contact time in CAMHS and is higher in subjects without pharmacological treatment and in those without a diagnosis recorded in their medical histories. In contrast, it is lower in subjects diagnosed with psychosis, bipolar disorder or psychotic depression, eating disorders, mental retardation, and neurodevelopmental disorders excluding ADHD.

Several studies also indicate that greater adherence is directly related to longer contact time with the service [25]. These data support the need to develop strategies to engage adolescents and youth during the early stages of care [12, 26].

Our results suggest that subjects with apparently less severe disorders have a higher risk of withdrawing from the service before the transition. Data are quite consistent with the results of the TRACK study [6].

It could be thought that the fact of not receiving pharmacological treatment and not belonging to the diagnostic groups indicated above imply “less severity”, but our results are still alarming for this reason. It is known that the indicated prevention for adolescent subjects with subthreshold symptoms or vulnerability can modify the expected trajectory of psychiatric disorders [27]. In this sense, the fact that such a large volume of patients with an established diagnosis abandoned care is a warning of a real risk to health. In fact, there is evidence that episodes of a common mental disorder in adolescence often precede mental disorders in young adults [28].

This study has several limitations that should be noted. First, it is a retrospective study, which means that some data is limited. In this sense, it has not been possible to know and analyze the reasons for dropping out of the service that the patients themselves may have stated. However, the fact that it is an epidemiological and retrospective study has allowed the inclusion of all eligible subjects without the bias of an informed consent for the prospective studies. Second, although the patient sample is large, it is limited to one territory of the Spanish state and it should be extrapolated to other settings with this consideration. Third, given that the sources of the information are clinical records, there could be a bias in the diagnoses by assuming the diagnosis established by the physicians and not by standardized clinical scales.

## Conclusions

From the clinical perspective, this study shows the existence of a very high risk of dropping out of the services during the period prior to transition age. The results suggest that the problem of continuity of care during the age of transition should be considered with a broader perspective than the phenomenon of changing services itself. Additionally, the results support the hypothesis that patients at this age have care needs that are not being adequately covered with traditional models of care. Nevertheless, future studies are necessary to analyze the reasons that lead adolescents to drop out of services early due to the impact that this behavior can have on their own health.

## Supplementary Information

Below is the link to the electronic supplementary material.

Supplementary file1 (DOCX 102 KB)

Supplementary file2 (DOC 39 KB)

## Data Availability

Database of the study and analysis are available under request.
