# Supplementary material for: The black hole of the transition process: dropout of care before transition age in adolescents
Source: Eur Child Adolesc Psychiatry. 2022 Jan 20;32(7):1285–95. doi: 10.1007/s00787-021-01939-8 (PMC10276128; doi:10.1007/s00787-021-01939-8)
Supplement: Supplementary file 1 — Supplementary file1 (DOCX 102 KB) [file 787_2021_1939_MOESM1_ESM.docx]

**Annex 1: Detailed diagnosis grouping**

| **Diagnostic groups** | **ICD-10 diagnostic codes** |
| --- | --- |
| Serious and enduring mental disorders | Including schizophrenia, psychotic disorders: F20 - F29.  Bipolar affective disorder: F30 - F31. Cyclothymia: F34.0.  Depression with psychosis: F32.3, F33.3.  Psychotic disorder due to use of cannabinoids: F12.5. Psychotic disorder due to multiple drug use and use of other: F19.5. |
| Emotional/neurotic disorders | Including non-psychotic depression F32, F33, F34.1. F38 (excluded depression with psychosis F32.3, F33.3)  Anxiety disorders: F40, F41, F44, F45, F48.  Obsessive-compulsive disorder: F42.  Reaction to severe stress, and adjustment disorders: F43.  Emotional disorders with onset specific to childhood: F93.  Disorders of social functioning with onset specific to childhood and adolescence: F94.  Other behavioral and emotional disorders with onset usually occurring in childhood and adolescence: F98, F99 |
| Eating disorders | Including anorexia nervosa, bulimia nervosa, atypical eating disorder: F50 |
| Neurodevelopmental disorders (excluded ADHD) | Including Mental retardation: F70 - F79.  Disorders of psychological development: F80, F83, F84, F88. |
| Attention deficit hyperactivity disorder (ADHD) | Attention deficit hyperactivity disorder: F90 |
| Conduct disorders | Including conduct disorder: Conduct disorder: F91.  Mixed disorders of conduct and emotions: F92. |
| Substance use disorders | Substance use: F10 - F19.  (Excluded psychotic disorder due to use of cannabinoids: F12.5 and Psychotic disorder due to multiple drug use and use of other F19.5) |
| Emerging personality disorders | Including specific personality disorder: F60.  Mixed and other personality disorders: F61  Habit and impulsive disorders: F63  Gender identity disorders: F64.  Psychological and behavioral disorders associated with sexual development and orientation: F66. |
| None diagnosis | Without principal and secondary diagnosis |
