# Supplementary material for: The black hole of the transition process: dropout of care before transition age in adolescents
Source: Eur Child Adolesc Psychiatry. 2022 Jan 20;32(7):1285–95. doi: 10.1007/s00787-021-01939-8 (PMC10276128; doi:10.1007/s00787-021-01939-8)
Supplement: Supplementary file 2 — Supplementary file2 (DOC 39 KB) [file 787_2021_1939_MOESM2_ESM.doc]

Annex 2. Description of ascertaining and grouping comorbid diagnosis

| **Original comorbid diagnostic groups** | **Diagnostic group selected for the study** |
| --- | --- |
| Serious and enduring mental disorders and Emotional/neurotic disorders.  Serious and enduring mental disorders and Neurodevelopmental disorders (excluded ADHD).  Serious and enduring mental disorders and Substance use disorders.  Serious and enduring mental disorders and Conduct disorders.  Serious and enduring mental disorders and Emerging personality disorders | Serious and enduring mental disorders |
| Emotional/neurotic disorders and Conduct disorders.  Obsessive-compulsive disorder (F42) and Attention deficit hyperactivity disorder (ADHD).  Obsessive-compulsive disorder (F42) and Substance use disorders. | Emotional/neurotic disorders |
| Eating disorders and emotional/neurotic disorders.  Eating disorders and Conduct disorders.  Eating disorders and Substance use disorders.  Eating disorders and Emerging personality disorders. | Eating disorders |
| Neurodevelopmental disorders (excluded ADHD) and Emotional/neurotic disorders.  Neurodevelopmental disorders (excluded ADHD) and Conduct disorders.  Neurodevelopmental disorders (excluded ADHD) and Substance use disorders.  Neurodevelopmental disorders (excluded ADHD) and Emerging personality disorders.  Pervasive developmental disorders (F84) and Attention deficit hyperactivity disorder (ADHD).  Mental retardation and Attention deficit hyperactivity disorder (ADHD) | Neurodevelopmental disorders (excluded ADHD) |
| Attention deficit hyperactivity disorder (ADHD) and Emotional/neurotic disorders.  Attention deficit hyperactivity disorder (ADHD) and Disorders of psychological development (F80, F83, F88).  Attention deficit hyperactivity disorder (ADHD) and Conduct disorders. Attention deficit hyperactivity disorder (ADHD) and Substance use disorders.  Attention deficit hyperactivity disorder (ADHD) and Emerging personality disorders. | Attention deficit hyperactivity disorder (ADHD) |
| Conduct disorders and Substance use disorders. | Conduct disorders |
| Substance use disorders and Emotional/neurotic disorders. | Substance use disorders |
| Emerging personality disorders and Emotional/neurotic disorders.  Emerging personality disorders and Conduct disorders.  Emerging personality disorders and Substance use disorders. | Emerging personality disorders |
